# JAK signaling regulates germline cyst breakdown and primordial follicle formation in mice

**DOI:** 10.1242/bio.029470

**Published:** 2017-12-14

**Authors:** Kun Huang, Ye Wang, Tuo Zhang, Meina He, Guanghong Sun, Jia Wen, Hao Yan, Han Cai, Changfu Yong, Guoliang Xia, Chao Wang

**Affiliations:** 1 Department of Physiology, State Key Laboratory of Agrobiotechnology, College of Biological Sciences, China Agricultural University, Beijing 100193, China; 2 Animal Disease Control and Prevention Center of Shapotou District, Zhongwei, Ningxia 755000, China

**Keywords:** JAK signaling, Primordial follicle formation, Germline cyst breakdown, Pregranulosa cell proliferation, Germ cell loss

## Abstract

In female mammals, primordial follicles consist of two types of cells, namely, oocytes and pregranulosa cells that surround the oocytes. The size of the primordial follicle pool determines the reproductive ability of female mammals. However, the underlying mechanisms controlling primordial follicle assembly remain unclear. In this study, we show that oocyte-derived Janus kinase (JAK) signaling is vital for germline cyst breakdown and primordial follicle formation *in vitro*. JAK2 and JAK3 activity is increased while germline cysts are breaking down. Inhibition of either JAK2 or JAK3 prevents germline cyst breakdown and primordial follicle formation. We further show that specific suppression of JAK2 delays germ cell loss through the downregulation of p53, but has no influence on pregranulosa cell proliferation. Alternatively, specific inhibition of JAK3 decreases pregranulosa cell proliferation by downregulating Notch2 signaling, implying that JAK3 acts on pregranulosa cells by controlling the extracellular secretion of oocyte-derived factors. In summary, our results indicate that JAK signaling contributes to germline cyst breakdown and primordial follicle formation by regulating oocyte loss and pregranulosa cell proliferation in the fetal mouse ovary. Our findings contribute to a better understanding of the molecular mechanism of mammalian folliculogenesis.

## Abstract (precis)

**Summary:** This research clarifies the important and distinct roles of JAK family members in primordial follicle formation of female mammals, and further explore the regulation mechanism of primordial follicle formation.

## INTRODUCTION

The initial establishment of the primordial follicle pool dictates the reproductive ability and duration of female mammals (Pepling and Spradling, 2001; Pepling, 2006). In mice, primordial germ cells (PGCs) migrate from the extraembryonic ectoderm to the genital ridge and then divide by mitosis with incomplete cytokinesis, followed by germline cyst formation at 10.5 days post coitus (dpc) (Mork and McLaren, 1981). Starting at 13.5 dpc, PGCs undergo meiosis, differentiate into oocytes, and arrest at the diplotene stage at approximately 17.5 dpc (Pepling and Spradling, 1998; Wang et al., 2015). Then, some oocytes in cysts survive and are enclosed by pregranulosa cells to form the primordial follicle, whereas others undergo apoptosis (Pepling, 2006). Temporally and quantitatively synchronous development between germ and pregranulosa cells is indispensable for well-organized folliculogenesis (Wang et al., 2017; Lei et al., 2006). Nevertheless, the molecular mechanisms of cyst breakdown and primordial follicle assembly remain unknown.

In mammals, the Janus kinase/signal transducer and activator of transcription (JAK/STAT) pathway is a pleiotropic cascade that transduces developmental signals (Schindler and Darnell, 1995). The JAK family includes four members: JAK1, JAK2, JAK3 and Tyk2. These proteins are responsible for transferring extracellular signals generated by polypeptide ligands, such as growth factors or cytokines, into cells via auto phosphorylation. Subsequently, activated JAKs phosphorylate downstream effectors, including their major substrates, the STATs (Shuai et al., 1993). Activated JAK stimulates cell proliferation, differentiation, migration and apoptosis. Impaired JAK/STAT signaling causes inflammatory disease, erythrocytosis, gigantism and leukemia (Igaz et al., 2001; O'Shea et al., 2002). In *Drosophila*, JAK/STAT participates in regulating somatic stem cells to prevent precocious germline stem cell differentiation (Wang et al., 2008), suggesting that the JAK/STAT pathway regulates the cross-talk between germ cells and somatic cells in oogenesis. In mice, STAT3 is localized in the cytoplasm of oocytes, wherein it possibly functions during cyst breakdown and primordial follicle assembly (Chen et al., 2007; Zhao et al., 2016). These findings imply that JAK is potentially a vital factor for regulating primordial follicle formation.

The Notch signaling pathway is a classical signaling pathway that regulates cell differentiation, proliferation, and apoptosis (Artavanis-Tsakonas et al., 1995, 1999). In mice, deletion of *Notch2* or its oocyte-derived ligand *Jagged1* led to loss of germline cyst breakdown and primordial follicle formation through the regulation of ovarian somatic cell development (Xu and Gridley, 2013; Vanorny et al., 2014). Interestingly, the relationship between Notch signaling and the JAK/STAT pathway in regulating *Drosophila* follicle development was reported by Assa-Kunik et al. (2007), who showed that *Delta* (NOTCH ligand)-mutant follicles failed to form stalk cells on their anterior side, coupled with antagonistic interactions with the JAK/STAT pathway. However, whether the findings in flies are relevant to mammals must be determined. The aim of this study was to uncover the possible role of JAK/STAT in murine folliculogenesis *in vitro*, and to identify its relationship with Notch signaling during this process.

## RESULTS

### The expression of JAK family members in the fetal and neonatal ovary

**Fig. 1.**
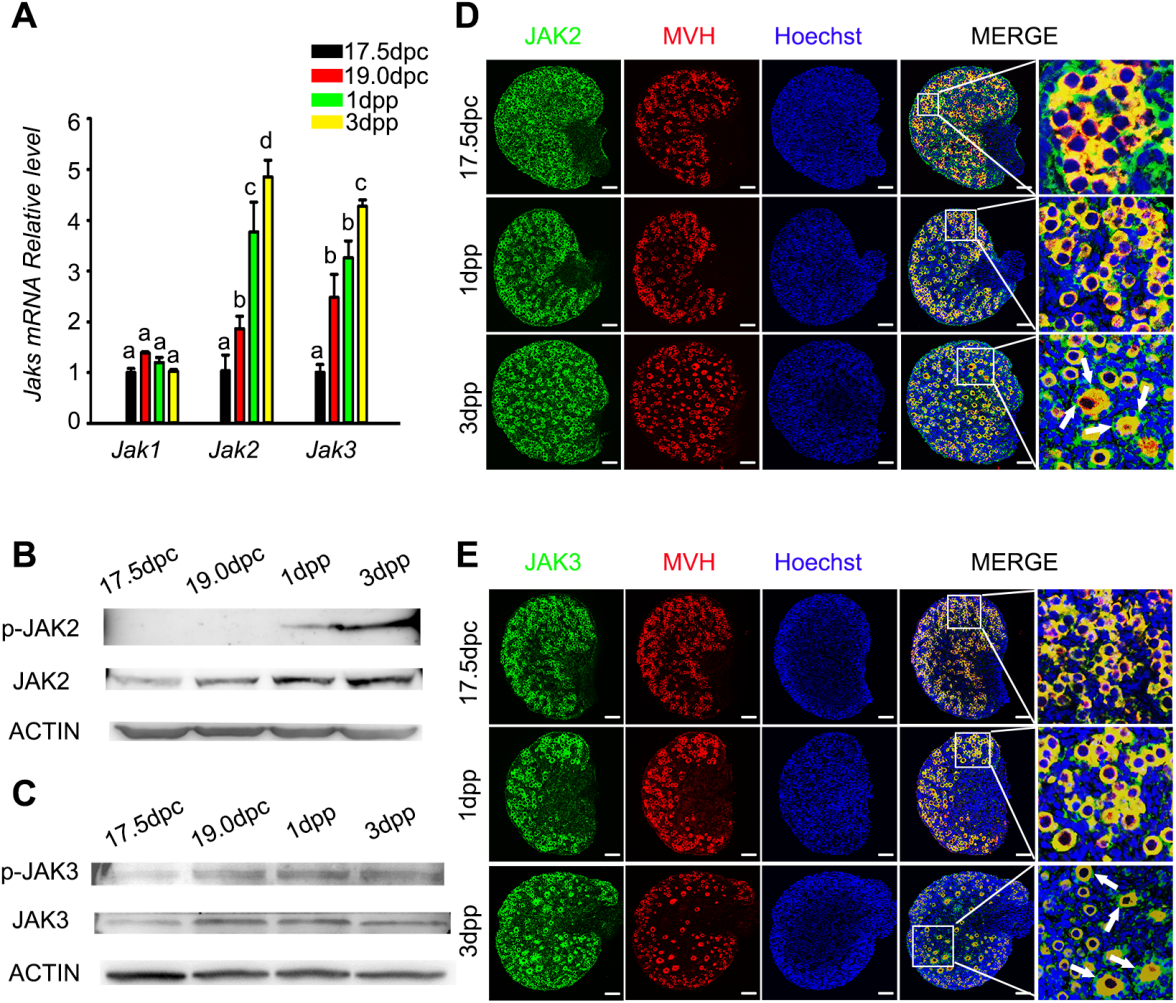
**Expression of JAK family members in the fetal and neonatal ovary.** (A) Expression of *Jak* family members from 15.5 dpc to 3 dpp ovaries. Transcripts were analyzed by real-time PCR (*n*=10). Statistical significance is indicated by different letters (a-d) (ANOVA and Holm–Sidak test). Error bars indicate the mean±s.d. from three different experiments. (B,C) Western blot analysis of JAK2 and JAK3 expression patterns in perinatal ovaries. β-ACTIN served as a loading control (*n*=15). (D,E) Ovaries were immuno-stained for JAK2 or JAK3 (green) and the oocyte marker MVH (red) at 17.5 dpc, 1 dpp, and 3 dpp. Hoechst (blue) was used to stain nuclear DNA. Scale bar: 100 μm. (*n*=3) Data are representative of three independent experiments.

To investigate whether JAK signaling is involved in germline cyst breakdown and primordial follicle formation, we first measured the mRNA expression of JAK family members in perinatal ovaries. As shown in Fig. 1A, *Jak1* mRNA levels were low, and there were no significant differences from 17.5 dpc to 3 days postpartum (dpp). Contrarily, both *Jak2* and *Jak3* mRNA levels increased in a time-dependent manner during cyst breakdown and the establishment of the primordial follicle pool. Western blot analysis showed that both total and phosphorylated levels of JAK2 and JAK3 protein increased from 17.5 dpc to 3 dpp (Fig. 1B,C).

The subcellular localization of JAK2 and JAK3 was detected by immunofluorescence. Ovarian sections from 17.5 dpc, 1 dpp and 3 dpp were labeled with antibodies against JAK2 or JAK3 and, MVH, a cytoplasmic oocyte marker. The results showed that both JAK2 and JAK3 were colocalized with MVH and mainly expressed in the oocyte cytoplasm (Fig. 1D,E). In addition, JAK2 and JAK3 were detected in the cytoplasm of cuboidal granulosa cells in growing follicles from 3 dpp ovaries (Fig. 1D,E, white arrows). Furthermore, ovaries at 17.5 dpc, 1 dpp, and 3 dpp were immuno-stained with antibodies against JAK2 or JAK3 and, FOXL2, a nuclear marker of granulosa cell. The results showed that JAK2 and JAK3 were not colocalized with FOXL2, but were observed in the cytoplasm of cuboidal granulosa cells in growing follicles at 3 dpp ovaries (Fig. S1A,B, white arrows). These results indicate that JAK2 and JAK3 may be involved in primordial follicle formation in the mouse ovary.

### Suppression of either JAK2 or JAK3 disrupts germline cyst breakdown and primordial follicle assembly

**Fig. 2.**
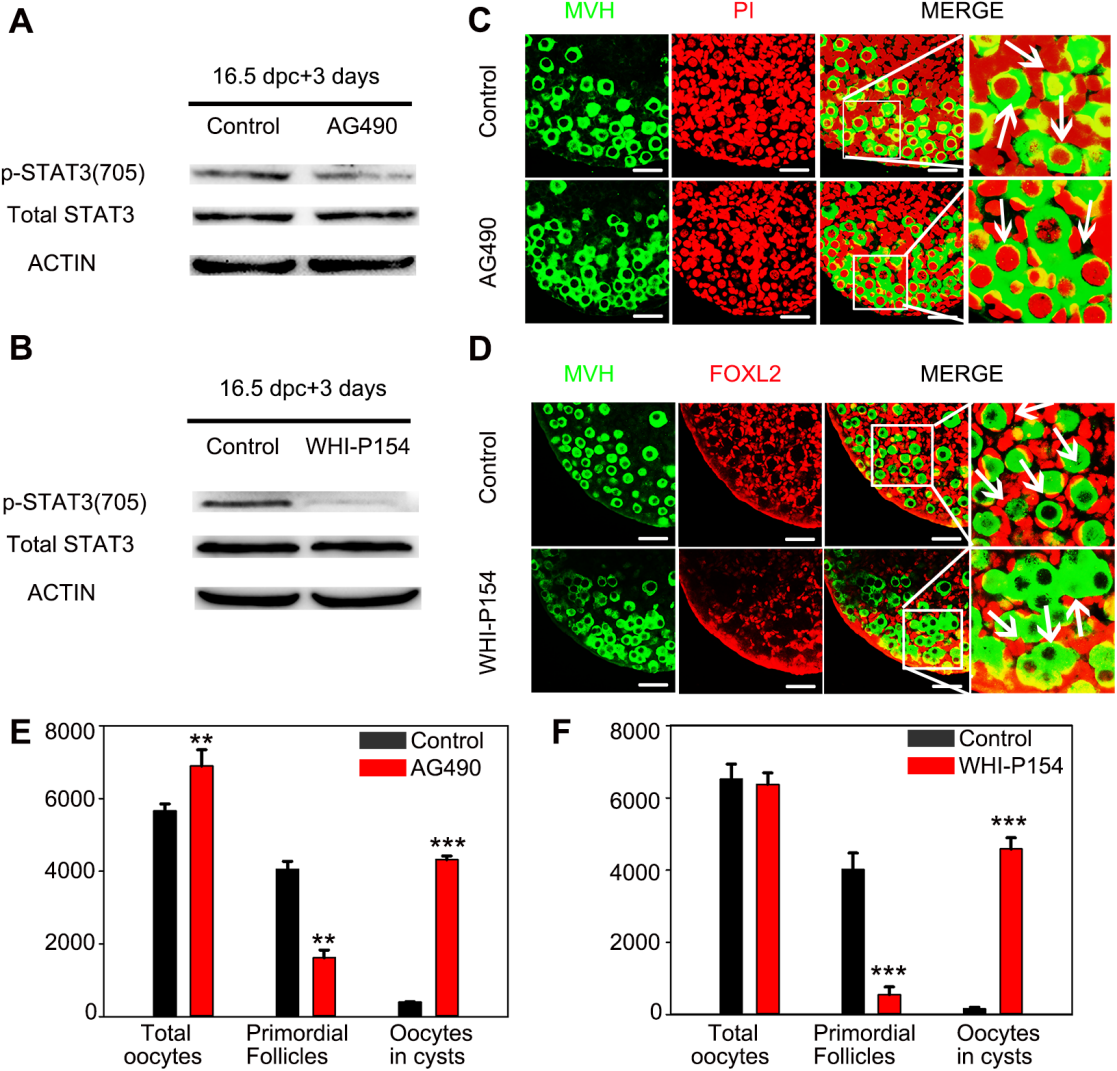
**Suppression of JAK2 or JAK3 disrupts germline cysts breakdown and primordial follicle assembly.** (A,C,E) Ovaries at 16.5 dpc were cultured with AG490 (20 μM) *in vitro*. (B,D,F) Ovaries at 16.5 dpc were cultured with WHI-P154 (15 μM) *in vitro.* (A,B) After 3 days of culture, phospho-STAT3 and total STAT3 expression levels in AG490-, WHI-P154, or control-treated ovaries were analyzed by western blot. β-ACTIN served as a loading control (*n*=10). (C,D) After 7 days of culture, AG490-treated ovarian sections were immunolabeled with MVH (green) and the nuclear marker propidium iodide (PI, red); WHI-P154-treated ovarian sections were immunolabeled with MVH (green) and the pregranulosa cell marker FOXL2 (red). Scale bar: 50 μm. (E,F) The number of total oocytes, primordial follicles and oocytes in cysts was quantified. **P<0.01 and ***P<0.001 (*t*-test), control versus treated ovaries (*n*=6). Data are representative of three independent experiments. Error bars indicate the mean±s.d. from three different experiments.

To determine whether JAKs play vital roles in germline cyst breakdown and primordial follicle formation, 16.5 dpc fetal ovaries were collected and cultured in different media *in vitro*. The ovaries were cultured for 3 or 7 days with or without AG490 or WHI-P154, which are specific inhibitors of JAK2 and JAK3, respectively (Linwong et al., 2005; Li et al., 2013). Western blot analysis revealed that Y705 phosphorylation of STAT3, the main downstream target protein of JAK/STAT signaling, was noticeably inhibited by AG490 or WHI-P154 (Fig. 2A,B). The ovaries from 16.5 dpc mice were cultured for 7 days with AG490 or WHI-P154, and the phenotypic differences were observed by immunofluorescence. Cyst breakdown was markedly inhibited in JAK inhibitor-treated ovaries, and the majority of oocytes remained wrapped in cysts (Fig. 2C,D). The number of oocytes within germline cysts was increased, while the number of primordial follicles was obviously decreased compared to that in the control group (Fig. 2E,F). Interestingly, the total number of oocytes in control ovaries was the same as that in WHI-P154-treated ovaries (Fig. 2F); however, the total number of oocytes was significantly higher in AG490-treated ovaries than in control ovaries (Fig. 2E). Taken together, these results suggest that JAK signaling plays vital roles in the establishment of the primordial follicle pool.

### Attenuation of JAK3 signaling, not JAK2 signaling, prohibits ovarian pregranulosa cell proliferation in perinatal ovaries

**Fig. 3.**
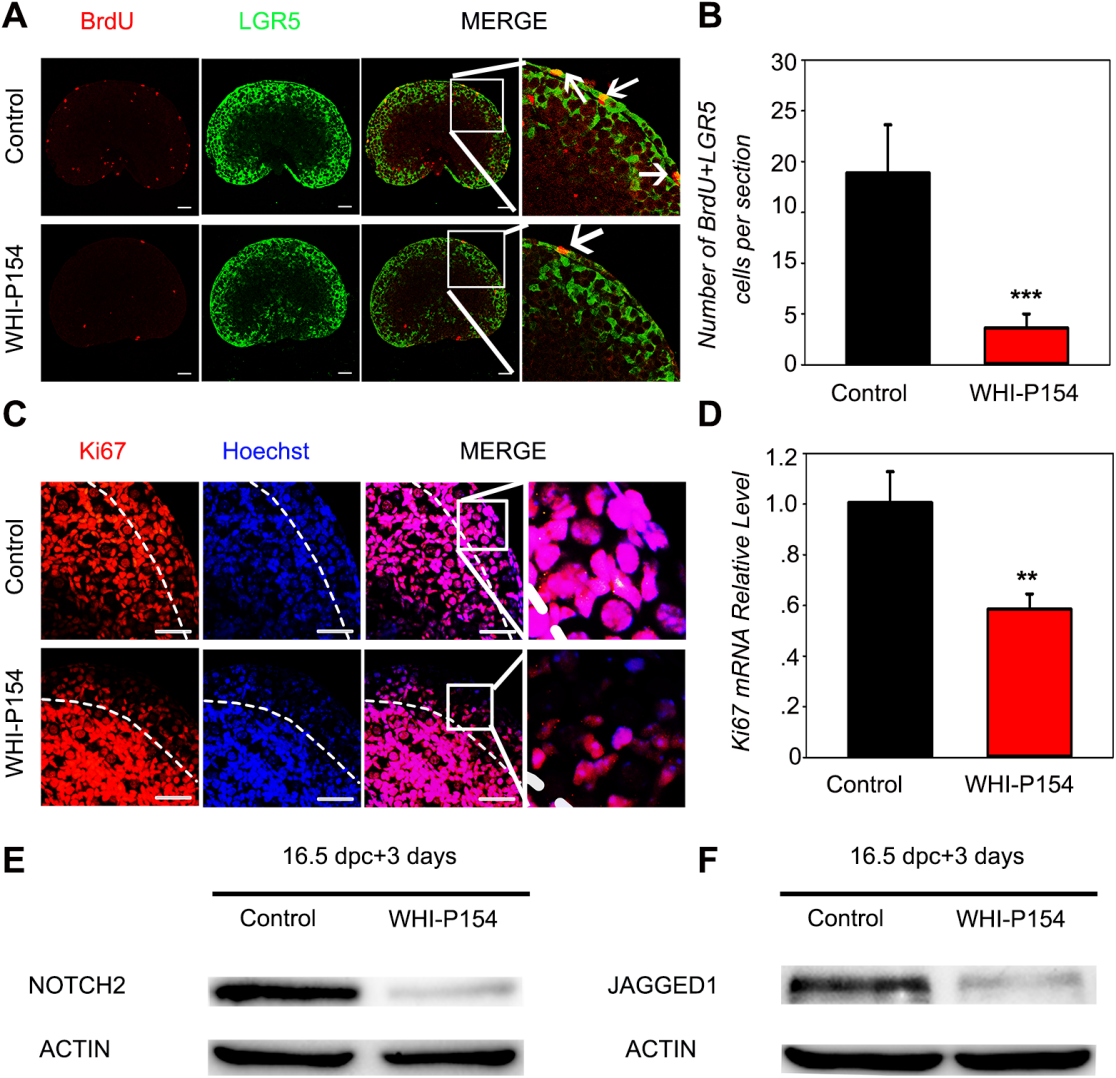
**Attenuation of JAK3 signaling prohibits ovarian pregranulosa cell proliferation in perinatal ovaries.** (A,B) LGR5-EGFP ovaries at 16.5 dpc were cultured with WHI-P154 (15 μM) for 3 days *in vitro* (n=6). (A) Ovarian sections were immunolabeled with GFP (green) and BrdU (red, white arrows). Scale bar: 100 μm. (B) The number of BrdU+/LGR5+ cells was quantified. ***P<0.001 (*t*-test), control versus treated ovaries. (C-F) CD1 ovaries at 16.5 dpc were cultured with WHI-P154 (15 μM) for 3 days *in vitro* (*n*=10). (C) Ovarian sections were immunolabeled with MKi67 (red). Hoechst (blue) was used to stain nuclear DNA. Scale bar: 50 μm. (D) *MKi67* expression in WHI-P154-treated ovaries and controls. **P<0.01 (*t*-test). (E,F) NOTCH2 and JAGGED1 expression in WHI-P154-treated ovaries and controls. β-ACTIN served as a loading control. Data are representative of three independent experiments. Error bars indicate the mean±s.d. from three different experiments.

To investigate how JAK signaling regulates primordial follicle formation, 16.5 dpc *Lgr5-*EGFP (Leucine rich repeat containing G protein coupled receptor 5-enhanced green fluorescence protein) ovaries were treated with AG490 or WHI-P154. Rastetter et al. (2014) showed that LGR5-positive cells in ovarian epithelial and cortical regions represent proliferating cells that subsequently differentiate into forkhead box L2 (FOXL2)-positive granulosa cells that assemble into the primordial follicle. After 3 days of culture, BrdU incorporation assays revealed that most of the LGR5-positive pregranulosa cells from the control ovarian epithelium were positive for BrdU. Conversely, the number of pregranulosa cells positive for both LGR5 and BrdU was dramatically decreased in WHI-P154-treated ovaries compared to control ovaries (Fig. 3A), and the number of LGR5+/BrdU+ pregranulosa cells in the ovarian epithelial and cortical regions was quantified (Fig. 3B). Moreover, we labeled the ovarian sections with an antibody against MKi67, a marker of proliferating cells. The immunofluorescence results showed that MKi67 expression was obviously downregulated in WHI-P154-treated ovaries compared to control ovaries (Fig. 3C), and the *MKi67* mRNA level was also markedly decreased (Fig. 3D). However, the number of LGR5+/BrdU+ pregranulosa cells was not significantly different after JAK2 inhibition compared to control treatment (Fig. S2A,B).

Previous studies have reported that *Notch2* deletion in granulosa cells results in the inhibition of LGR5-positive cell proliferation and differentiation and impaired fertility, accompanied by the formation of multi-oocyte follicles (Feng et al., 2016). We investigated whether Notch signaling is affected by JAK signaling. Fetal ovaries at 16.5 dpc were cultured *in vitro* for 3 days following treatment with the JAK2 inhibitor AG490 or the JAK3 inhibitor WHI-P154. Western blot analysis showed that NOTCH2-JAGGED1 signaling was obviously downregulated after JAK3 inhibition (Fig. 3E,F). However, NOTCH2 and JAGGED1 protein levels were not significantly changed following the suppression of JAK2 activity (Fig. S3A,B). The results indicate that JAK3 signaling, not JAK2 signaling, controls pregranulosa cell proliferation in the murine ovarian epithelium.

### JAK2 signaling regulates germline cyst breakdown and germ cell loss through p53

**Fig. 4.**
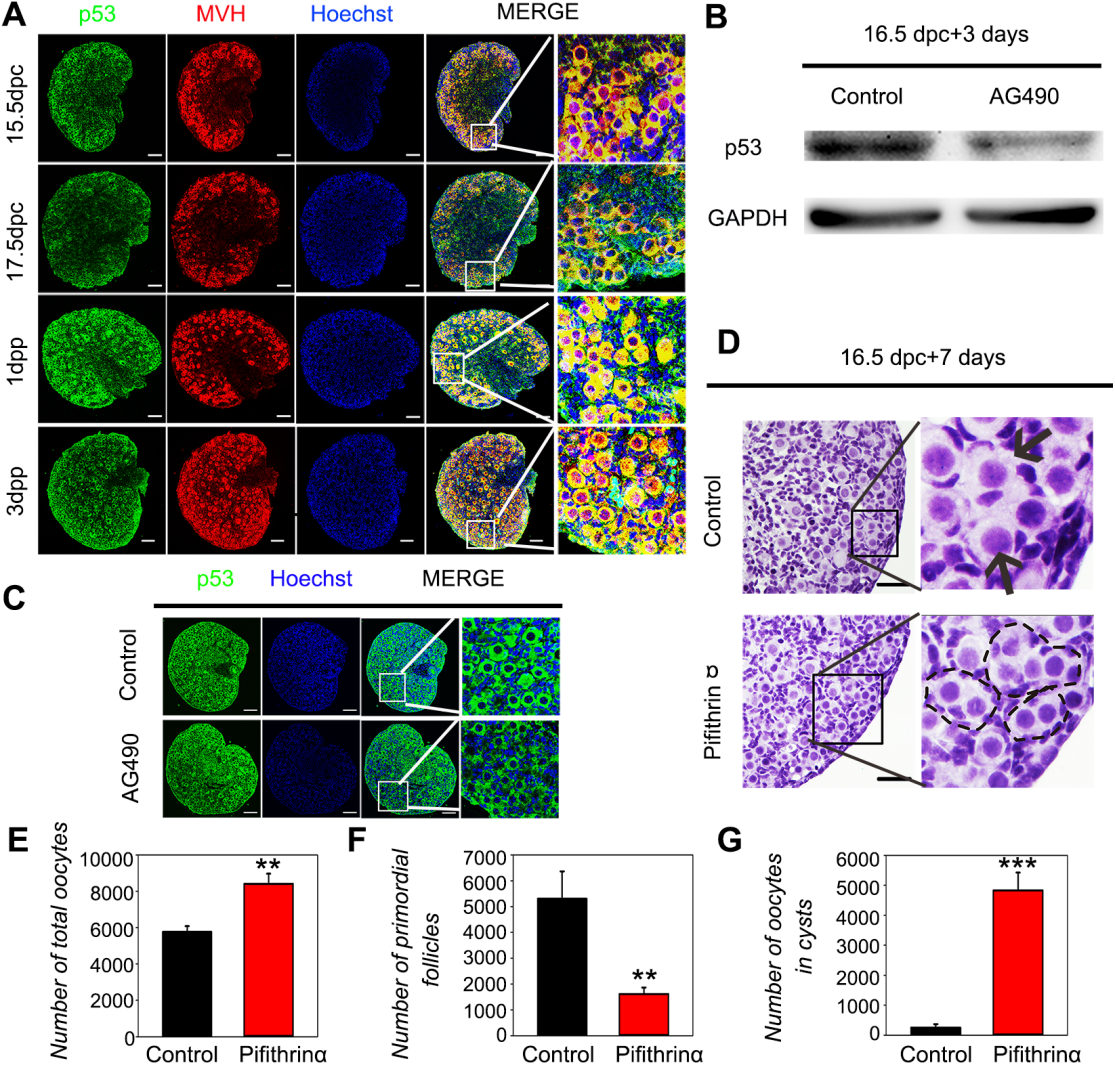
**JAK2 signaling regulates germline cyst breakdown and germ cell loss through p53.** (A) Ovaries were immunostained for p53 (green) and MVH (red) at 15.5 dpc, 17.5 dpc, 1 dpp, and 3 dpp. Nuclei were visualized with Hoechst (blue). Scale bar: 100 μm (*n*=3). (B) CD1 ovaries at 16.5 dpc were cultured with AG490 (20 μM) for 3 days *in vitro* (*n*=15). Western blot analysis showed the downregulation of p53 in AG490-treated ovaries. GAPDH served as a loading control. (C-G) CD1 ovaries at 16.5 dpc were cultured with pifithrin-α (10 μM) for 7 days *in vitro* (*n*=6)*.* (C) After 7 days of culture, immunofluorescence analysis revealed the weak expression of p53 (green) in AG490-treated ovaries. Hoechst (blue) was used to identify nuclear DNA. Scale bar: 100 μm. (D) Hematoxylin staining analysis showed that cyst breakdown was inhibited by pifithrin-α in the mouse ovary (black dotted lines), whereas primordial follicles assembled in control ovaries (black arrows). Scale bar: 50 μm. (E,F,G) The number of total oocytes, primordial follicles and oocytes in cysts was quantified. **P<0.01 and ***P<0.001 (*t*-test), control versus treated ovaries. Data are representative of three independent experiments. Error bars indicate mean±s.d.

We speculated that JAK2 might regulate cyst breakdown and germ cell loss by controlling the expression of p53, a crucial transcriptional factor that regulates apoptosis, cell cycle arrest, and genetic stability (Levine, 1997). We examined the *in vivo* p53 expression pattern during primordial follicle assembly, and western blot analysis showed that p53 protein levels were increased (Fig. S4). Immunofluorescence revealed that p53 was expressed in the oocyte cytoplasm from 15.5 dpc to 3 dpp (Fig. 4A).

Next, we cultured 16.5 dpc mouse ovaries *in vitro* for 3 days with AG490, and western blot analysis showed that proliferation cell nuclear antigen (PCNA) protein levels were similar to those in control ovaries, and the immunofluorescence results were consistent (Fig. S5A,B), suggesting that the increased number of germ cells was not due to proliferation. Furthermore, Bcl-2-associated X (BAX) protein was downregulated in AG490-treated ovaries, indicating that inhibiting JAK2 arrested germ cell apoptosis (Fig. S6). More interestingly, p53 protein levels were obviously downregulated in AG490-treated ovaries (Fig. 4B). Immunofluorescence results confirmed that suppressing JAK2 led to a decrease in p53, especially in germline cyst structures (Fig. 4C).

To better clarify the role of p53 in primordial follicle formation, 16.5 dpc fetal ovaries were cultured *in vitro* with the p53 inhibitor pifithrin-α for 3 days. Effective p53 inhibition was confirmed by the downregulation of *p21* mRNA compared to the control (Fig. S7). Subsequently, 16.5 dpc ovaries were treated with pifithrin-α for 7 days, and ovarian histology was evaluated. The germline cysts in the control ovaries broke down to form primordial follicles; conversely, cyst breakdown was delayed in the pifithrin-α-treated group (Fig. 4D). Quantification of oocytes revealed that pifithrin-α-treated ovaries had significantly fewer primordial follicles and, consequently, more oocytes remaining within cysts compared to control ovaries (Fig. 4F,G). Moreover, the total number of oocytes was higher in pifithrin-α-treated ovaries (Fig. 4E). These results suggest that p53 is important for germline cyst breakdown and germ cell loss, and JAK2 signaling regulates cyst breakdown and germ cell loss through p53.

## DISCUSSION

**Fig. 5.**
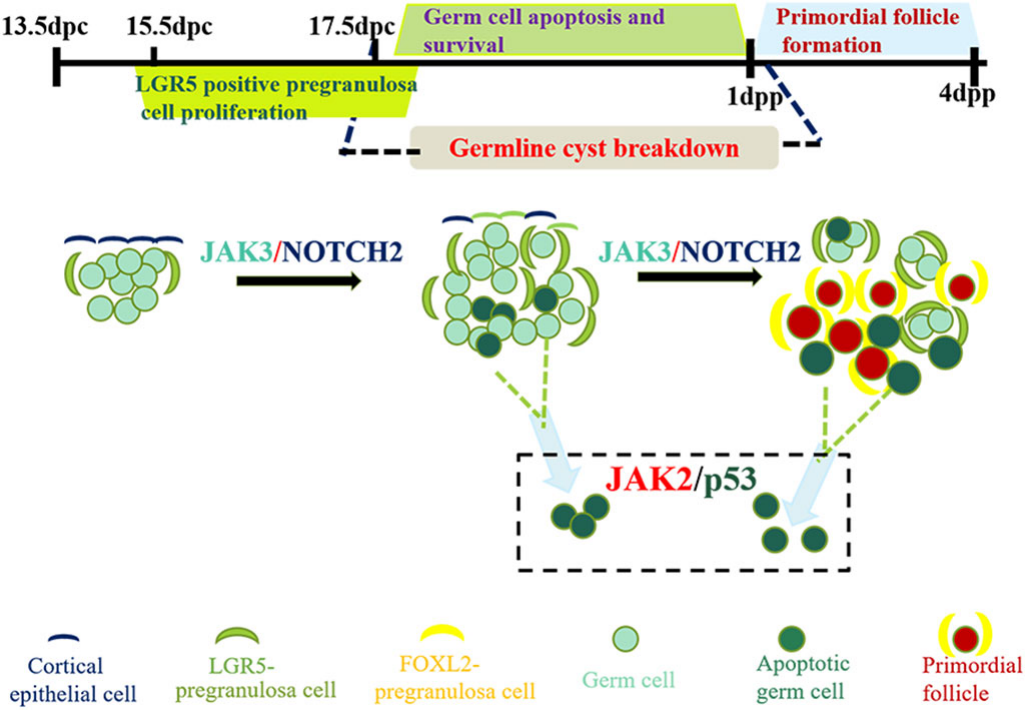
**Model of JAK signaling in germline cyst breakdown and primordial follicle formation.** Oocyte-derived Janus kinase (JAK) signaling is vital for germline cyst breakdown and primordial follicle formation *in vitro*. JAK2 and JAK3 activity shows an increasing trend while germline cysts break down. Pregranulosa cells surround oocytes to form primordial follicles at 4 dpp when JAK signaling peaks. We show that JAK2 and JAK3 signaling regulates germ cell loss and pregranulosa cell proliferation, respectively, to participate in primordial follicle formation.

According to previous reports, JAK signaling acts on somatic cells and maintains germline cell differentiation synchronously during follicle development in *Drosophila* (Wang et al., 2008). In mammals, the primary functions of JAK family members are in the immune system (Shuai and Liu, 2003). In this study, we provide evidence that JAK2 and JAK3 signaling, probably by regulating germ cell loss and pregranulosa cell proliferation, respectively, participates in murine primordial follicle formation *in vitro* (Fig. 5).

### The role of JAK3/STAT3 in the proliferation of ovarian epithelial LGR5-positive cells during primordial follicle formation

In mammals, an important part of primordial follicle formation is the communication between oocytes and pregranulosa cells (Pepling, 2006). Notch signaling, the medium for communicating between two types of cells, plays an important role in cyst breakdown and follicle assembly (Xu and Gridley, 2013). However, the mechanism by which Notch signaling is regulated needs further exploration. STAT3 has been extensively reported as an oocyte-derived protein in perinatal and adult mouse ovaries that is essential during mammalian development; mice with a targeted disruption of *Stat3* die in the embryonic stage (Murphy et al., 2005; Takeda et al., 1997), and activated STAT3 suppresses the differentiation of mouse embryonic stem cells (Raz et al., 1999). Subsequently, it was reported that STAT3 dictates primordial follicle formation by controlling the transcription of factors secreted by oocytes, including GDF9, BMP15 and JAGGED1, which results in changes in the levels of the somatic cell-derived factor NOTCH2 at the translational level (Zhao et al., 2016). Meanwhile, JAK3 is a vital factor in the regulation of NOTCH2 expression and LGR5-positive pregranulosa cell proliferation (Feng et al., 2016). Our results show that inhibiting JAK3 leads to a significant downregulation of STAT3. Therefore, JAK3 possibly regulates NOTCH2 expression by activating STAT3, which results in pregranulosa cell proliferation and primordial follicle assembly.

### JAK2, which controls germ cell loss by regulating BAX expression, may participate in cyst breakdown and primordial follicle formation

Cysts break down into single oocytes in two days following birth. During this process, two-thirds of oocytes die by programmed cell death, and only one third of oocytes survive to be subsequently surrounded by flat pregranulosa cells (Pepling and Spradling, 2001). Bcl-2 family members were identified as pivotal regulators of germ cell loss (Cory and Adams, 2002). However, the regulators of the Bcl-2 gene family are unknown, and the mechanism of this regulation during germ cell loss has not been elucidated. Previous reports have clarified that abnormalities in JAK2/STAT3 signaling are associated with oncogenesis in several types of cancer. Constitutive activation of STAT3 correlates with cell proliferation in non-small-cell lung cancer (Alvarez et al., 2006) and pancreatic cancer (Sahu and Srivastava, 2009); conversely, inhibiting JAK2/STAT3 signaling arrests the growth of primary human cancer cells (Chiarle et al., 2005) and induces colorectal cancer cell apoptosis by modulating the Bcl-2 gene family, promoting the loss of mitochondrial transmembrane potential and increasing the levels of reactive oxygen species (Du et al., 2012). We found that JAK2 inhibition leads to an increased number of total germ cells following the marked downregulation of BAX expression in perinatal ovaries. Therefore, JAK2 may participate in cyst breakdown and primordial follicle formation by regulating germ cell loss during mouse ovarian development. However, the molecular mechanism by which JAK2 controls germ cell loss *in vitro* is unclear.

### The possible mechanism by which JAK2 regulates p53 expression depends on MDM-p53 ubiquitylation and degradation during the period of primordial follicle formation

According to previous research, p53 is a crucial transcription factor in cell cycle progression, cell survival, and apoptosis (Levine, 1997). Wild-type p53 induces apoptosis in MDAH 2774 and Caov-3 ovarian cancer cells with high levels of JAK2 phosphorylation (Reid et al., 2004). In addition, Nakatake et al. (2012) showed that the JAK2^V617F^ mutation affects the p53 response to DNA damage through the accumulation of Murine double minute 2 (MDM2), an E3 ubiquitin ligase that binds to p53 and promotes its proteosomal degradation in myeloproliferative neoplasms. In ascertaining the relationship between p53 and JAK2 in ovarian development, we found that JAK2 regulated p53 expression during cyst breakdown and germ cell loss and that p53 expression was markedly weaker in the cyst structures of JAK2-inhibited ovaries. The results indicate that p53 is regulated by JAK2 signaling in the perinatal mouse ovary. However, further studies are necessary to determine whether JAK2 regulates p53 via MDM2.

### Differences in the regulatory mechanisms governing JAK2 and JAK3 signaling in primordial follicle formation

Although the present study was not extensive enough to systematically explain the relationship between JAKs and primordial folliculogenesis, our findings provide the primary principles of JAK2 or JAK3 signaling in germ cells or pregranulosa cells. First, JAKs are possibly stimulated by various membrane receptors and their extracellular ligands. For example, insulin-like growth factor activates either JAK2 or STAT3 (Himpe and Kooijman, 2009). Jones and Pepling (2013) reported that Kit-Kit ligand signaling regulates germ cell apoptosis during primordial follicle formation, in which KIT autophosphorylation activates several downstream cascades, including the JAK/STAT pathway (Schlessinger, 2000). Some cytokines, such as IL-2, IL-4, and IL-7, mainly activate JAK3 (Nosaka et al., 1995), although the role of cytokines in murine primordial follicle formation has not been adequately studied. Second, JAK possibly regulates different intracellular factors to elicit diverse functions because different STATs selectively respond to JAKs (O'Shea et al., 2015). STAT3 is the only STAT that has been previously studied in primordial follicle formation. In our study, we found that JAK2 and JAK3 have diverse roles in oocyte and pregranulosa cell development, probably because individual JAK signaling responds to various extracellular factors or activates different STATs.

Taken together, our results clarify that JAK2 or JAK3 signaling regulates folliculogenesis in mice by influencing various intracellular mediators, as shown *in vitro*. However, the JAK/STAT regulatory network in folliculogenesis must be further studied in genetically modified animals and using *in vivo* assays.

## MATERIALS AND METHODS

### Ethical approval

The animal studies were conducted using protocols approved by the Institutional Animal Care and Use Committee of China Agricultural University (License No. SKLAB2017-01-01).

### Animals

The Lgr5-EGFP-ires-CreERT2 (Lgr5-KI) reporter mice have been described in detail previously (Ng et al., 2014). Male and female CD1 mice at 6-8 weeks were purchased from the Laboratory Animal Center of the Institute of Genetics in Beijing. Animals were maintained in China Agricultural University with a ratio of 16 h light:8 h darkness at 25°C, with free access to food and water. Matings were accomplished in the next morning with vaginal plug detection. Mice with a vaginal plug were considered 0.5 dpc.

### Chemicals

AG490 (S1143), WHI-P154 (S2867) and Pifithrinα (S2929) were purchased from Selleck Chemicals (Texas, USA). All reagents were diluted with DMSO. AG490 (5 mg) was in 100 mM stock solution, WHI-P154 (5 mg) was in 50 mM stock solution and Pifithrin-α was in 10 mM stock solution.

### Ovaries culture

Fetal ovaries from 16.5 dpc mice were dissected in PBS under sterile conditions. Ovaries were cultured in 1 ml DMEM/F12 mixture (GIBCO, Life Technologies) with 6-well culture plates (NEST, Jiangsu, China) at 37°C, 5% CO_2_. Half of the medium was replaced every other day.

### Immunofluorescence and Hematoxylin staining

Collected ovaries were fixed overnight at 4°C in 4% paraformaldehyde for immunohistochemistry. Samples were treated through an ethanol series and xylene, infiltrated with paraffin wax, and sliced into 5 μm thick sections. Sections were dewaxed in xylene and rehydrated in ethanol series, and then were dyed with hematoxylin for 1 min. For immunofluorescence staining, sections were heated on high power for 4 min once and on low power for 4 min three times in 0.01 M sodium citrate (pH 6.0) for antigen retrieval. Then sections were blocked for 1 h in Immunol Staining Blocking Buffer (P0102) at room temperature. Primary antibody were diluted in PBS and sections were incubated overnight at 4°C (antibody information is listed in Table S1). Then samples were incubated with secondary antibodies, either Alexa Fluor 488-conjugated, 555-conjugated anti-mouse, anti-goat, anti-rabbit or anti-chicken (1:100; Invitrogen), for 80 min at 37°C. Hoechst 33342 (B2261; Sigma) or PI (Propidium Iodide Solution, 421301, BioLegend) were used for nuclear DNA. Sections were coverslipped with anti-fade fluorescence mounting medium (Applygen, Beijing, China). The images were acquired on a confocal microscope (Eclipse, Nikon, Japan) and analyzed by the NIS-Elements BR 3.2 software.

### BrdU assay

For detection of pregranulosa cell proliferation, bromodeoxyuridine (BrdU, B5002; Sigma) was added to the medium to 10 μM and incubated for 1 h at 37°C. The ovaries were fixed in 4% paraformaldehyde overnight at 4°C for immunofluorescence analysis.

### Oocyte counting of whole ovary

In our study, every six to fifteen ovaries were randomly allocated into the control or treated group in one experiment. Ovaries were sliced into 5 μm thick sections, and every five sections were counted, considering that the diameter of a follicle is about 20-25 μm. Number was counted respectively and multiplied by five to represent each index for one ovary.

### RNA isolation and quantitative real-time PCR

Total RNA were extracted using TRIzol (Invitrogen, Life Technologies, USA) from ovaries according to the manufacturer's protocol. Real-time PCR assays were performed using SYBR Green PCR master mix and mouse β-actin served as the internal control. The primer sequences are listed in Table S2.

### Western blot analysis of lysates

Ovaries were lysed in WIP (Beijing Cell Chip Biotechnology Co., Beijing, China) according to the manufacturer's protocol. Equal amounts of protein from lysates were subjected to 10% SDS–PAGE and then transferred to PVDF membranes (Millipore). PVDF membranes were blocked for 1 h with 5% (v/v) non-fat milk in phosphate buffered saline (PBS) with 0.1% (v/v) Tween 20 at room temperature. The blots were incubated with primary antibodies overnight at 4°C (antibody information is listed in Table S1). The blots were incubated with horseradish peroxidase-linked secondary antibodies (ZB-2301, ZB-2305 from ZSGB-BIO, Beijing, China) for 1 h at room temperature. Protein were detected by a Super Signal Chemiluminescent Detection System (34080; Thermo Scientific). The blots were acquired by Chemiluminescence image system (Tanon 5200, Beijing, China). The level of β-ACTIN and GAPDH were used as an internal control.

### Statistical analysis

Data were presented as means±s.d. in triplicate. Data were analyzed either by *t*-test or ANOVA. If a significant *F* ratio was confirmed by ANOVA, data of the related groups were analyzed by the Holm–Sidak test. *P* values <0.05 were considered statistically significant.

## Supplementary Material

Supplementary information
