# Supplementary material for: JAK signaling regulates germline cyst breakdown and primordial follicle formation in mice
Source: Biol Open. 2017 Dec 14;7(1):bio029470. doi: 10.1242/bio.029470 (PMC5827266; doi:10.1242/bio.029470)
Supplement: Supplementary information [file biolopen-7-029470-s1.pdf]

Supplementary Materials

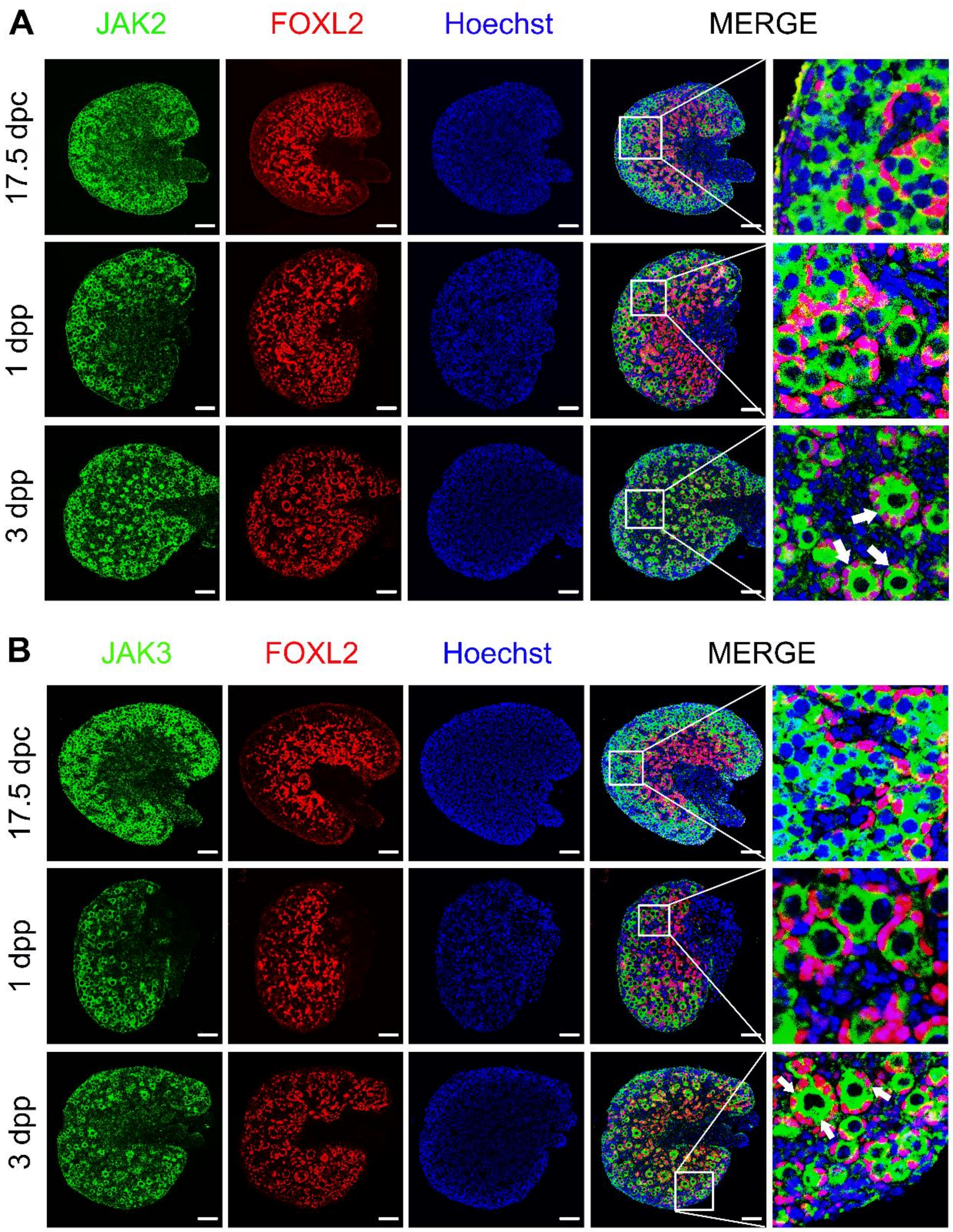

Fig. S1. Expression of JAK family members in the fetal and neonatal ovary. (A, B) Ovaries were immuno-stained for JAK2 or JAK3 (green) and FOXL2 (red), a nuclear marker of granulosa cells at 17.5 dpc, 1dpp, and 3 dpp. Hoechst (blue) was used to stain nuclear DNA. Scale bar: 100 µm. Data are representative of three independent experiments.

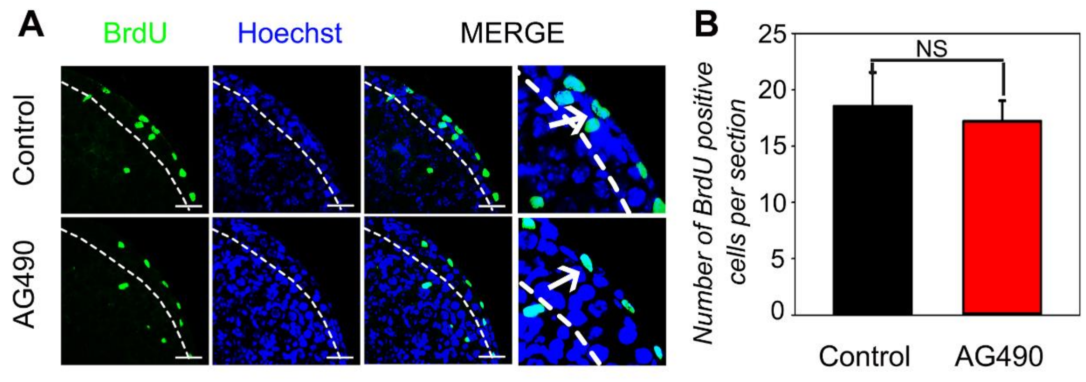

Fig. S2. The pregranulosa cell proliferation was not controlled by JAK2. (A) CD1 ovaries at 16.5 dpc were cultured with AG490 (20 μM) *in vitro* for 3 days. Ovarian sections were immunolabeled with BrdU (green). Hoechst (blue) was used to identify the nuclear DNA. Scale bars: 50 μm; (B) The number of BrdU-positive cells was quantified. Representative data of three separate experiments are shown as means ± s.d. of triplicate assays. No statistical significance is shown (NS). *t*-test, control versus treated ovaries.

Biology Open • Supplementary information

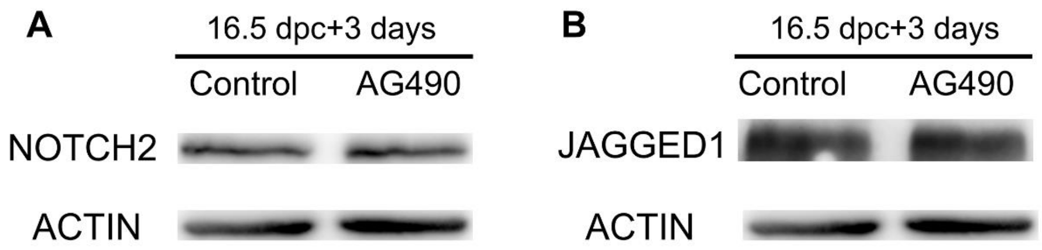

Fig. S3. Notch signaling was not significantly changed following suppression of JAK2 activity. CD1 ovaries from 16.5 dpc were cultured with AG490 (20 μM) for 3 days *in vitro*. (A, B) Western blot analysis of NOTCH2 and JAGGED1 expression in AG490-treated ovaries and controls. β-ACTIN served as a loading control. Data are representative of three independent experiments.

Biology Open • Supplementary information

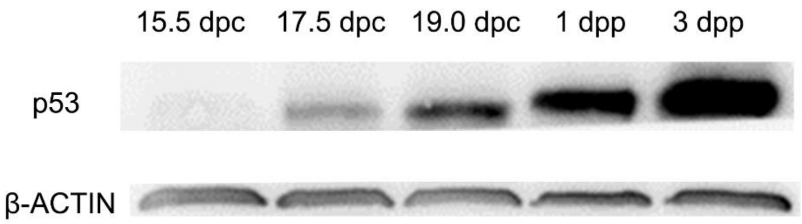

Fig. S4. p53 protein expression in fetal and neonatal ovaries. Ovarian proteins from 15.5 dpc to 3 dpp were analyzed by western blot. β-ACTIN served as a loading control. Data are representative of three independent experiments.

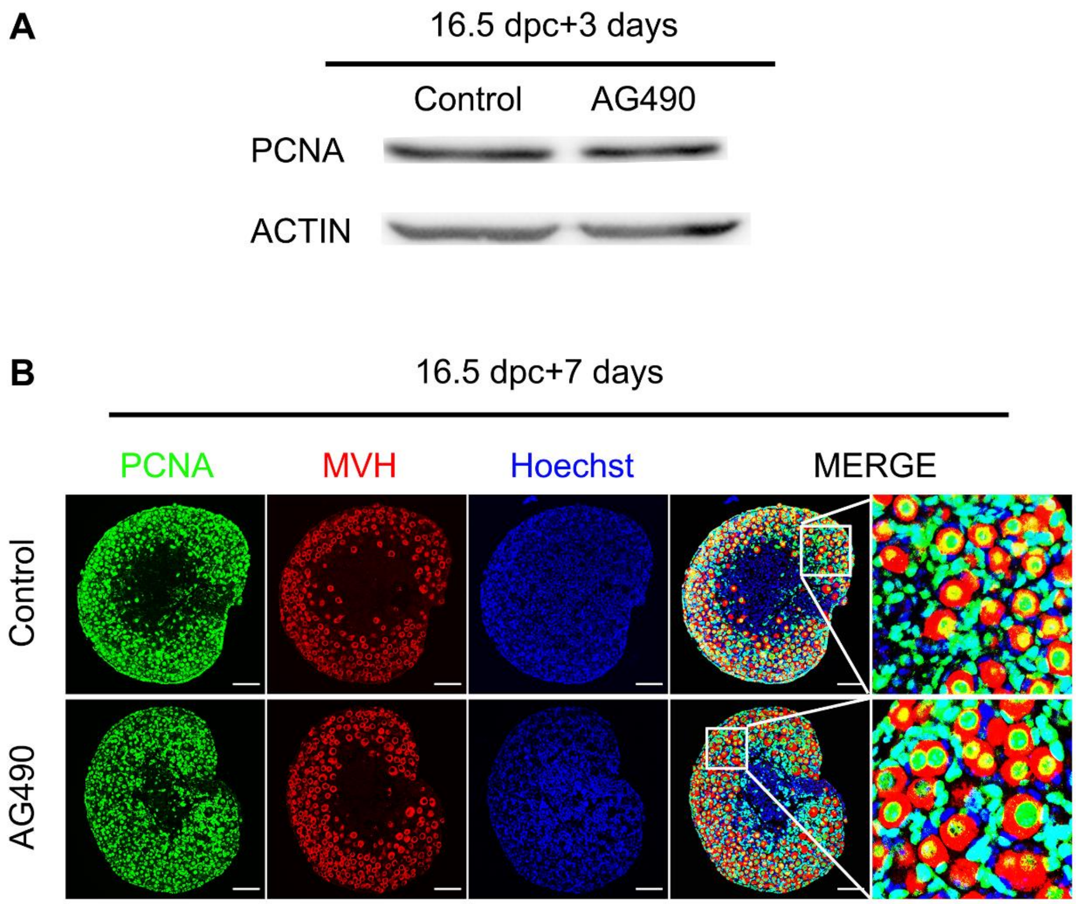

Fig. S5. Inhibition of JAK2 led to germ cell increase without impacting PCNA expression. (A) CD1 ovaries at 16.5 dpc were cultured with AG490 (20 μM) for 3 days *in vitro*. Expression of PCNA in AG490-treated ovaries and controls. β-ACTIN served as a loading control; (B) CD1 ovaries from 16.5 dpc were cultured with AG490 (20 μM) for 7 days *in vitro*. Ovarian sections were immunolabeled with PCNA (green) and with MVH (red). Hoechst (blue) was used to identify the nuclear DNA. Scale bars: 100 μm. Data are representative of three independent experiments.

Biology Open • Supplementary information

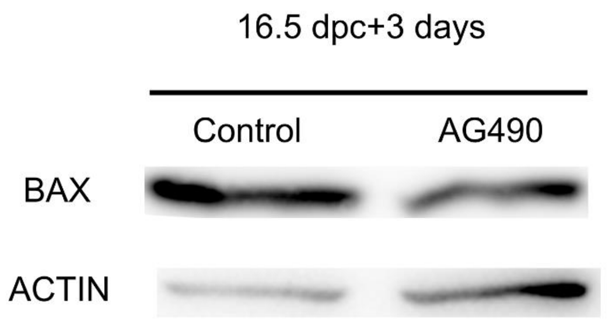

Fig. S6. Inhibition of JAK2 decreased BAX protein expression. CD1 ovaries at 16.5 dpc were cultured with AG490 (20 μM) for 3 days *in vitro*. Expression of BAX protein in AG490-treated ovaries and controls. β-ACTIN served as a loading control. Data are representative of three independent experiments.

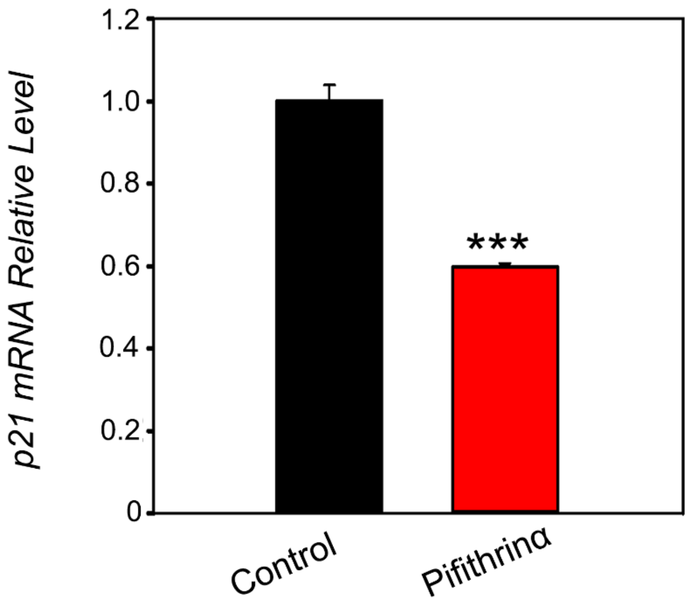

Fig. S7. The suppression efficiency of p53 inhibitor pifithrinα in cultured ovaries was detected by p21 expression downregulation. CD1 ovaries at 16.5 dpc were cultured with AG490 (20 μM) for 3 days *in vitro*. Total ovarian RNA was extracted and transcripts were analyzed by real-time PCR. Representative data of three separate experiments are shown as means ± SD of triplicate assays. Statistical significance is shown. ***$p < 0.001$, (*t*-test), control versus treated ovaries.

Biology Open • Supplementary information

Table S1. List of primary antibodies used in the immune research

Antibody	Catalog Code	Source	Host	Dilution	
				IF	WB
p-JAK2	ab32101	Abcam	rabbit		1:500
JAK2	ab108596	Abcam	rabbit	1:200	1:500
p-JAK3	sc-16567	Santa cruz	rabbit		1:500
JAK3	ab91206	Abcam	mouse	1:200	1:500
p-STAT3	#9145	CST	rabbit		1:500
STAT3	#9139	CST	rabbit		1:500
GFP	ab13970	Abcam	chicken	1:200	
FOXL2	IMG-3228	Novus	goat	1:200	
p53	ab28	Abcam	mouse	1:200	1:500
MVH	ab13840	Abcam	rabbit	1:200	
MVH	ab27591	Abcam	mouse	1:200	
BrdU	G3G4	DSHB	mouse	1:200	
MKi67	9129S	CST	rabbit	1:200	
PCNA	sc-56	Santa cruz	mouse	1:200	1:500
NOTCH2	#5732	CST	rabbit		1:500
JAGGED1	sc-8303	Santa cruz	rabbit		1:500
BAX	ZS5260	ZSGB-BIO	rabbit		1:200
β-ACTIN	CW0096M	CWbiotech	mouse		1:1000
GAPDH	AM4300	Ambion	mouse		1:1000

Biology Open • Supplementary information

Table S2. List of primers used in qRT-PCR

Genes	Forwards (5'……3')	Reverse (5'……3')
Jak1	CTCGCTCGTCCTTTCGGTG	TGTCCCTGAAAGCAGACTGG
Jak2	GCGACGGGAACAAGATGTGA	TTGTAAGGCAGGCCATTCCC
Jak3	CCCTGACGTAGCGGACTTT	AATGACAATCGCTGGGGAGG
p21	CCACCACCAAGCCATTCCAT	CACCACACTATCCTGGGCAT
Mki67	GACTGCGAGCTTCACCGAG	AGGCAGCTGGATACGAATGTC
Actin	GTGACGTTGACATCCGTAAAGA	GCCGGACTCATCGTACTCC
